# *Holding open spaces to explore beyond*: Toward a different conceptualization of specialization in high-performance sport

**DOI:** 10.3389/fpsyg.2023.1089264

**Published:** 2023-03-02

**Authors:** Veronique Richard, John Cairney, Carl T. Woods

**Affiliations:** ^1^School of Human Movement and Nutrition Sciences, The University of Queensland, St Lucia, QLD, Australia; ^2^Institute for Health and Sport, Victoria University, Melbourne, VIC, Australia

**Keywords:** specialization, elite sports, ecological dynamics, holistic development, possibility

## Abstract

This conceptual analysis aims to challenge the state of high-performance sport by questioning the concept of *specialization*. To start, we offer a brief, but critical overview of what specialization currently entails. Then, shifting the paradigm, we suggest an expansion rather than a reduction of developmental possibilities once an athlete reaches the “top”. Specifically, rather than athletes conforming to national standards imposed by governing bodies about what it means to be “elite”, we suggest sport systems consider a *person-environment fit* approach to support ongoing development. Drawing on an ecological dynamics rationale and various socio-cultural theories, we explore how concepts such as *affordances* and *perspectives* can be harnessed to create a better “fit” between athletes’ action capabilities and the opportunities within their *broader environment*. Our conception of specialization requires moving away from a definition of success based on the accumulation of medals, toward one that accounts for the exploration and achievement of the *possible*. We argue that a person-environment fit welcomes diversity, so long as it sustains the person’s health, wellbeing, and performance. This, it is suggested, is about collectively holding open spaces for each other to explore beyond the constraints of high-performance sport, encouraging *all* to carry on their lives in directions meaningfully impactful for them. We conclude this conceptual analysis with a brief case example demonstrating what our theorizing could look like in practice.

## Introduction

According to the 1894 International Olympic Committee (IOC) statement: “the Olympic motto “Citius, Altius, Fortius”, is an expression of excellence, *not* the glorification of performance or victory. It is about *giving ones best*, improving and striving for perfection on a daily basis, both in the stadium *and in life*” (The Olympic Museum, emphasis added). Overtime, though, this ideal has given way to an exclusive “win at all costs” ethic, oft-guided through a “Faster, Higher, Stronger” mentality. Contrary to the IOC statement, it appears that the expression of “victory” has thus begun to dominate the narrative in high-performance sport (Douglas and Carless, 2006; Carless and Douglas, 2012). Perfection in the stadium, otherwise stated, seems to be all that matters.

This shift from a holistic developmental philosophy to a performance-centric one has undoubtedly shaped how sporting environments and systems are designed at the highest level (Vaughan et al., 2022). For instance, the pervasive myth that sport consists of prescribed regimental training creates environments where participants must adhere to rules, conform to techniques, and “fit-in” to be accepted and selected on the highest-level teams (Rothwell et al., 2020; Vaughan et al., 2022). While such compliance may benefit some, its exceptionalism risks rejecting diversity and inclusivity, leading to conformist behavior. Further, the “medals at all costs” mentality risks pressuring sport practitioners and scientists to find the most efficient methods to develop skill and athleticism, which potentially narrows focus, neglecting the development of attributes that are deemed less critical to those that are directly measurable and produce “wins” (Henriksen et al., 2020; Whitehead and Senecal, 2020). While sport has long been touted as a health enhancing pursuit (e.g., Eime et al., 2013; Oja et al., 2015), there is now increasing evidence of both the physical and mental health costs of the high-performance system (Peluso and Andrade, 2005; Douglas and Carless, 2015; Reardon et al., 2019; Franco et al., 2021). Specifically, athletes often risk being situated at the center of a toxic environment that erodes opportunities to explore interests, perhaps even beyond the sport itself (Whitehead and Senecal, 2020). Here, we push against such hyper-specialization in high-performance sport by asking whether experiencing a variety of enriching and meaningful activities – beyond sport – could promote athlete development, health, and performance. To us, sport – even at the highest levels – is more than winning medals. It can be an immensely rich environment replete with opportunities for athletes to carry on in various directions, pursuing things that spark their interest as they go (Woods and Davids, 2023).

Our conceptual analysis aims to challenge the state of high-level performance sport by questioning the concept of *specialization*. While the concept of *early* specialization is often criticized because of its negative physical, psychological and social impacts (Baker, 2003; Fraser-Thomas et al., 2008; Côté et al., 2009), few scholars have questioned the notion of *late* specialization. As depicted by various westernized sport pathways, specialization is often assumed to be what athletes must commit to, in order to achieve success (Côté et al., 2009; Gulbin et al., 2013; Way et al., 2016). The Canadian *Long-Term Athlete Development* framework (LTAD; Way et al., 2016) goes as far as classifying sports according to their *early* or *late* specialization requirements, clearly indicating that specialization *must* happen. But what does specialization actually entail?

In response to this question, we first explore the legacy of *deliberate practice* in sport, highlighting its consequences on current high-performance sport environments. We then briefly review the debate around early diversification and specialization, emphasizing the lack of consensus around sport developmental pathways and, thus, the need for a shift of paradigm. Building off an ecological approach, in appreciating the *organism-environment fit* (Gibson, 1979/1986), the second section presents an alternate conceptualization of specialization, one that holds open spaces for athletes to *explore beyond* the scope of their sport. To theoretically support our alternate concept, we lean on key ideas derived from an ecological dynamics perspective to inform the re-design of the sport ecosystem. The Gibsonian concept of “affordances” is specifically important here, used to illustrate how diverse opportunities for action can be designed *in* to high performance environments to support ongoing exploration beyond the sport itself. Surmising these ideas, in the last section, we suggest a change is needed in how “success” is measured in high-performance sport. Overall, by moving away from reductionist models of sport excellence toward a model that embraces complexity and diversity, our goal is to conceptualize specialization not as a narrowing in, but as an *opening up* to emergent possibility for exploration.

### The deliberate practice legacy

A salient example of a reductionist perspective lies in the concept of deliberate practice (Ericsson et al., 1993). Three criteria are often used to define deliberate practice: (i) *performers actively respond to a task with an explicit goal* (determined by an expert teacher); (ii) *immediate formative feedback is provided for each performance trial*; (iii) *opportunities to repeat the same or similar tasks must be afforded to the performer* (Ericsson, 2020). The assumption that the best performers engage in a greater quantity of deliberate practice than their least successful counterparts has been heavily investigated by the sport community (Baker and Young, 2014; Macnamara et al., 2016). Popular statements like, “ten thousand hours is the magic number of greatness” (Gladwell, 2008, p. 41), reinforce the already pervasive colloquialism that “practice makes perfect.” Despite Ericsson’s (2013, 2020) attempts to nuance “quantity” statements and meta-analytic results showing that the quantity of deliberate practice only slightly to moderately explains variance in sport performance (Macnamara et al., 2016), sport environments are still heavily “quantity-oriented” (Rees et al., 2016; Güllich et al., 2019). But at what cost?

An alarming example of the consequences of excessive “quantity-oriented” environments is highlighted in a study by Law et al. (2007). Comparing a group of elite (Olympic) rhythmic gymnasts against a group of sub-elite (international) peers, Law et al. (2007) revealed that by the age of 16, Olympic level gymnasts accumulated, on average, 18,835 training hours. From age 4 to 16, that means an average of 30.18 h/week of training. Unsurprisingly, elite gymnasts participated in less than two additional activities in this period. Such specialization also resulted in a significantly lower health and enjoyment when compared to their sub-elite counterparts, who “only” trained 6,686 h in the same period, and never won an international competition. Though, it is important to mention that with the third of the training time, a sustained health, and enjoyment of the experience, the international gymnasts were ranked first in their country and 13th in the world. It is, perhaps, legitimate to then question who the *real* winner *could* be.

Beyond its focus on quantity and repetition, and its consequences on health and enjoyment, the pervasiveness of the “deliberate practice legacy” in sport likely resides in the anchored assumption that hyper-specialization is, at some stage, the ultimate way to reach expertise (e.g., Rees et al., 2016; Güllich, 2017). Yet, in recent years, the accumulation of evidence showing that athletes struggle with mental health (e.g., Douglas and Carless, 2015; Poucher et al., 2019; Jewett et al., 2021) is challenging this assumption. While the incidence of mental health issues within the athletic population is similar to the one found in the general population (Gulliver et al., 2015; Rice et al., 2016), some elements of elite sport participation, such as overtraining and performance pressures, have been shown to increase an athlete susceptibility to mental health challenges (Rice et al., 2016; Van Slingerland, 2021). Specifically, following specialization, elite sport can become an “all-consuming” experience leading athletes to relinquish their personal autonomy and develop singular identities, which leaves them with few alternatives to shape their sense of self outside of sport (Douglas and Carless, 2009; Hughes and Leavey, 2012; Van Slingerland, 2021). Such anchored athletic identity can contribute to psychological distress and burnout (Cresswell and Eklund, 2007; Hughes and Leavey, 2012), leading scholars to question whether highly specialized sport environments are sustainable for athlete development (Gucciardi et al., 2017; Whitehead and Senecal, 2020). For example, the woman artistic gymnastics competition in the Tokyo summer Olympics, the woman figure skating event in the Beijing winter Olympics, as well as athletes withdrawing from high level competitions in a variety of sports suggests that we may have reached a breaking point. Indeed, while recognizing the complex and multidimensional factors that each contribute to mental health and wellbeing, here, we question whether competitive, high-performance sporting environments could be a contributing factor due to over-emphasis on quantity instead of diversity and balance (Brohm, 1978; Martinková, 2008).

### Diversification to counter specialization

To alleviate some of the negative consequences of the “deliberate practice legacy,” it has been proposed that children first be initiated to sport through deliberate play: a form of physical activity that is “intrinsically motivating, provide immediate gratification, and are specifically designed to maximize enjoyment” (Côté et al., 2013, p. 10). In line with this conceptualization, a debate has emerged between the effect of early specialization (e.g., deliberate, coach-led, structured practice in one sport) versus early diversification (e.g., deliberate play, peer-led, unstructured practice in multiple sports) on expertise development in sport (e.g., Coutinho et al., 2016a,b; Güllich, 2017). For instance, evidence suggests that specializing in a single sport at an early age (i.e., often before 12) can lead to negative physical (Bell et al., 2018), psychological and social consequences (Baker and Côté, 2006; Fraser-Thomas et al., 2008). On the other hand, early diversification is associated with expertise attainment through the development of motivation (Côté et al., 2009), perceptual-cognitive skill (Berry et al., 2008), adaptability (Rudd et al., 2020), and sport-specific creativity (Memmert et al., 2010). Nevertheless, other studies have shown that the difference between early “samplers” and “specializers” is often small and non-significant (Strachan et al., 2009; Coutinho et al., 2016b; Mosher et al., 2020; Aalberg et al., 2022). Moreover, some argue that the lack of consensus around the definition of “specialization” can cause confusion with “overtraining,” leading to discrepancies within the literature (Mosher et al., 2020). Aalberg et al. (2022) claim that the debate between early diversification and early specialization is “futile” because both can lead to excellence in sport. By comparing the environmental constraints for expertise development of world-class cross-country skiing versus free skiing, they found that while accumulated hours of training did not differ between groups, cross-country skiers heavily specialized into their sport after 16 years-old while the freestyle skiers kept engaging in unorganized training activities in other sports. They concluded by calling for a more individualized, depolarized, and culturally appropriate approach to expert development.

Perhaps, then, it is time to accept that there might be many varied approaches that can support expert performance (Carless and Douglas, 2012; Güllich et al., 2019). There may simply be no magic number or ideal pathway, hence, conceptualizing specialization based on number of hours and sole focus on sport may be misleading. By relying on aggregates, categorization and dichotomizations to describe and prescribe an athletes’ journey, developmental models may limit more than enhance the exploration of possibility (Baker et al., 2019). Following Sport New Zealand’s (2016–2020) advice: “To do a better job of identifying and developing our future high performers, […] we must let go of some stuff we know for sure that the research tells us just isn’t so” (p. 2). Inspired by an ecological dynamics rationale, and building on sociocultural theories, we next suggest an expansion rather than a reduction of developmental possibilities once an athlete reaches the supposed specialization stage. Specifically, instead of building sport systems that require athletes to conform to pre-determined standards, we suggest they consider the *person-environment fit*.

### Inside-out and outside-in approach

According to Baker et al. (2019), “[a]n individual’s capacity for positively interacting with the environments in which they find themselves may be the most significant constraint to maximizing their potential” (p. 32). How to facilitate positive interactions between an individual and environment, though, is a complex question. One way could be to create and maintain open spaces by offering malleable environments replete with varying opportunities for action. In youth sport, for example, to foster positive sport experiences, empirical evidence advises against environments that reduce individual opportunities for personal development (Erikstad et al., 2021). Elite sport systems do not frequently follow such advice. Once an athlete has reached the highest level in their sport, they are often faced with restrictive choices. Beyond sport-related choices, specialization risks reducing the possibilities for athletes to engage in other “extra-curricular” activities (e.g., Law et al., 2007), and may even force critical choices such as attending school, engaging in social gatherings, gaining professional experiences, or fulfilling familial responsibilities (e.g., Douglas and Carless, 2006; Henriksen et al., 2020; Whitehead and Senecal, 2020; Ni et al., 2021).

When some athletes are showing maladaptive responses to such specialized and overly constrained environments, such as motivational declines, raises in anxiety (Vaughan et al., 2022), burn out (Gustafsson et al., 2008), or drop out (Fraser-Thomas et al., 2008), internal causes are often identified, and resources are deployed to help the athlete develop skills to better “cope” with the external environment. A good example of this is how sport systems reacted to the increased in the number of athlete reporting mental health issues by developing clinical consensus (Reardon et al., 2019; Henriksen et al., 2020), creating mental health center (e.g., Canadian Centre for Mental Health and Sport; Van Slingerland et al., 2019) and increasing the number of psychology professionals working with athletes (Weir, 2018). While these initiatives are essential to enhance mental health literacy and awareness, they may not be complete (Purcell et al., 2019). Deploying what we will call here “inside-out” solutions may harbor what Dunwoody (2006) calls an “organismic asymmetry”; a trend in cognitive psychology to focus ones scale solely on the individual, neglecting the environment in which one inhabits. A public health way of framing this could be to question whether these approaches attempt to “fix” the individual after harm is done, rather than prevent harm in the first place by considering, not only the individual, but the environment and how it facilitates risk. This conceptual shift has influenced other areas such as workplace mental health programs and models (Harvey et al., 2014; Maslach, 2017).

To facilitate healthy interactions between an individual and the environment, we thus suggest that “inside-out” strategies be complemented with “outside-in” approaches. That is, considering and perhaps manipulating features of one’s environment to support opportunities for exploration, and thereby harbor a better “fit” between one’s action capabilities and the opportunities within their environment. Many have pointed to the importance of environmental factors (Ayala et al., 2022), ecological systems (Purcell et al., 2019), and even person-environment fit (Henriksen et al., 2020) to achieve positive and heathy sport experiences at the elite level. Yet, solutions to achieve this “fit” often target the micro-system (e.g., social support; Ayala et al., 2022) or are relying on coaches’ interventions/education (e.g., Reardon et al., 2019). Although coaches are indeed part of the sporting ecosystem, it is important to consider that they are often heavily constrained by sport socio-cultural norms and expectations and are also under the “medal at all costs” pressure (e.g., Jones et al., 2022). Hence, to create sustainable change, the paradigm shift must operate at the systemic level and be supported by a comprehensible framework to inspire stakeholders’ actions.

### Toward an ecological dynamics rationale of specialization

Inspired by the ongoing transition happening in the field of sports skill acquisition, we argue that *ecological dynamics* can offer a relevant framework to conceptualize what specialization could entail. Namely, skill acquisition research shifted from a computational (top-down) cognitive model, where skill relies on the acquisition of internal representations, to an embedded and embodied conceptualization, where skill emerges from a functionally constrained relationship between an individual and environment (Araújo and Davids, 2011). From a practical standpoint, this means that instead of implementing practice “drills” based on repeatable techniques to achieve an optimal movement pattern, practitioners are encouraged to design tasks that promote search and exploration, leading to the emergence of functionally adaptable performance solutions (Woods et al., 2020). Such a view moves away from a deterministic perspective of motor actions, which oft-fail to acknowledge the complex, reciprocal and non-linear relationships between a person and environment (Araújo and Davids, 2011). Therefore, to move away from the rigid, prescriptive and dualistic nature of the current specialization conceptualization, a similar shift toward relational mutuality, exploration, and complexity could facilitate re-orientation anchored around functional outcomes in sport and health. While ecological dynamics principles have mainly shown positive impacts on the emergence of rich and varied movement behaviors in sport (Hristovski et al., 2006; Pinder et al., 2012; Orth et al., 2018), we argue that in taking a socio-cultural perspective, concepts such as affordances, perspectives, actions, and possibilities can be harnessed to support the development of human behaviors through and beyond sport.

### Affordances

According to Gibson (1979/1986), “the affordances of the environment are what it offers the animal, what it provides or furnishes, either for good or for ill” (p. 127). In other words, affordances are opportunities for actions provided by a specific environment. An assumption that holds true in sport is that affordances are primarily associated to motor behaviors (Rietveld and Kiverstein, 2014). For instance, athletes’ perception of surfaces, events, or objects’ properties attract or invite various actions (Withagen et al., 2012), leading (or not) to functional performance solutions (e.g., Seifert et al., 2014). Following Heft (2013), we defined affordances as relational properties of a performer-environment system, which means their perception and actualization depends on a myriad of factors, such as performer characteristics, skills, intentions, and the socio-materiality of the environment (Reed, 1993; Araújo et al., 2019). Following this conceptualization, the acquisition of motor skill is no longer just about instructing athletes toward an “optimal” or “gold standard” technique, but about encouraging them to attend and respond to soliciting affordances within their environment (Araújo and Davids, 2011; Araújo et al., 2019).

Expanding the theory of affordances beyond the material, Rietveld and Kiverstein (2014) sought to “situate affordances in the context of a form of life” (p. 330). These authors argued that the human “form of life” is rich and resourceful, shaped not only by its material properties but also by a wide variety of socio-cultural practices. This opened affordances up to “the whole spectrum of social significance” (Gibson, 1979/1986, p. 127) which influence how skillful individuals engage, and thus act within a specific environment. In short, what individuals care about and what they prefer or need in a given sociocultural context, facilitate the detection of affordances, leading to an embodied and situated readiness for action (Rietveld and Kiverstein, 2014).

While engaging with a broad landscape of affordances is recognized for its positive outcomes (Rietveld and Kiverstein, 2014; Glăveanu, 2021), the consequences of socio-cultural norms that discourage interactions with affordances in certain *forms of life* is less extensively discussed. Reed (1993) uses the term *field of action* to discuss social processes that encourage or discourage intention toward specific affordances. Specifically, he defined the *field of promoted action* (FPA) as affordances which individuals are encouraged to actualize in a certain form of life and the *field of free action* (FFA) as affordances that individuals can realize on their own whilst being socially supported. Throughout individual’s developmental journey, Reed argues that the FFA should increase while the FPA should be refined. Taking sport as a specific form of life, it is critical to question whether this developmental trajectory from increased FFA to refined FPA is respected, and if not, what are the consequences. For instance, it could be relevant to interrogate what the impacts are of preventing athletes from trying new movement techniques or strategies because “it is not how we do things here,” restricting their engagement with nature (e.g., hiking a mountain) because they may injure themselves, refusing to let them interact meaningfully with opportunities afforded in other contexts because they must solely focus on training, or controlling their relationship with certain type of food or social opportunity because this is what it takes to win.

To elaborate further on this, in circus arts, the term “captured body” is used to refer to socio-cultural practices that restrict search, discovery and (inter)action with possibilities and have been associated to feelings of imprisonment, boredom and loss of agency (Lavers et al., 2019). These consequences of being bodily constrained by the cultural norms of the environment can be explained through radical embodied cognitive science (RECS). According to this conceptualization, “the mind is not solely located in the brain but also involves the body and the body’s situation in the environment” (Malinin, 2019, p. 2). Because the brain is attuned to what the body and the environment afford (Gallagher, 2015), restrictive environments leading to overly-prescribed ways of moving and acting in the world can impact the whole system. While sport research using RECS perspectives is expanding (Seifert et al., 2020; Schiavio et al., 2019), little is yet known about the physical, psychological, and social consequences of these “captured body” practices which may, or may not, disrupt the relationship between athletes and affordances that are significant to them. Hence, to initiate a healthier conceptualization of specialization, considering more deliberately how athletes experience the world may be critical.

**Figure 1:**
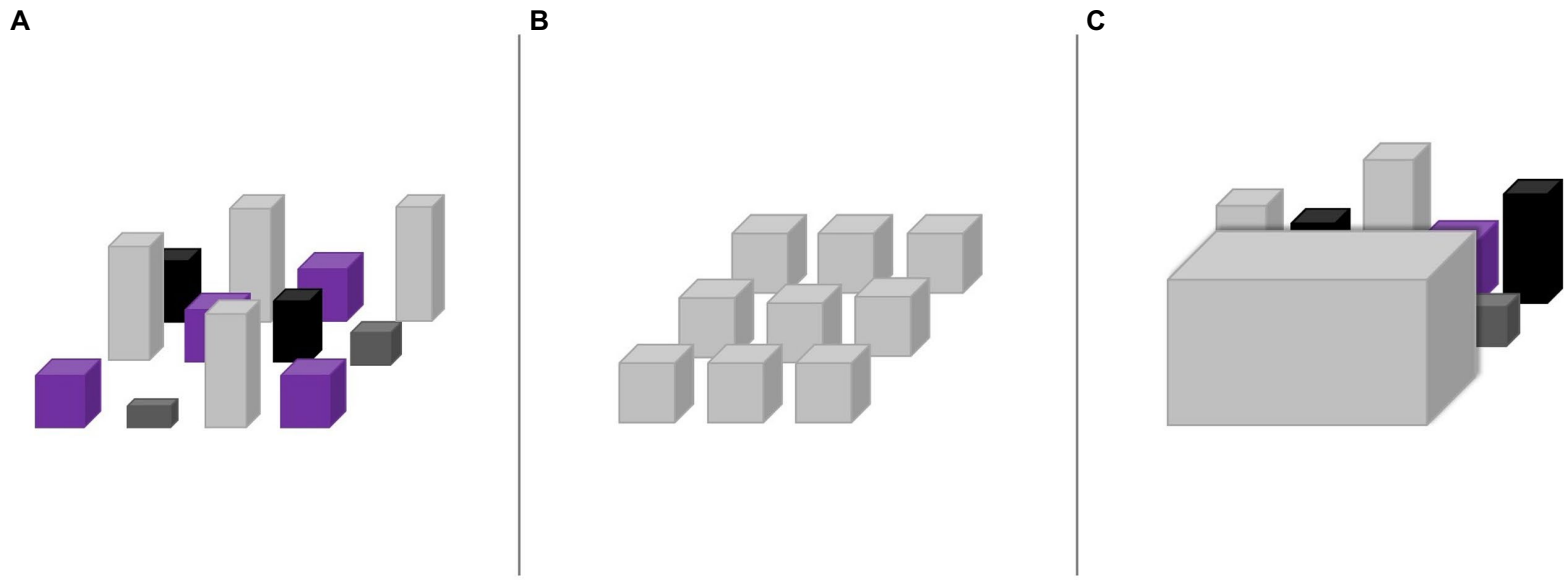
Landscapes of relevant affordances **(A)** meaningful, **(B)** unattractive, **(C)** unbalanced. In each image, the gray cubes represent the training in ones’ main sport while the other colors represent other interests, activities, responsibilities, etc. Inspired from Rietveld and Kiverstein (2014, p. 348).

While considerable efforts have been deployed to assess how athlete interact with their sport-specific environment, leading to the identification of meaningful affordances to be acted on (e.g., use of eye-tracking technology; Aksum et al., 2021), less attention has been directed toward understanding the consequences of athletes’ interactions with their *broader* environments. To initiate this investigation, we draw on de Haan et al. (2013) concept of the *enactive affordance-based model* to illustrate the person-world interactive dynamics and capture one’s responsiveness to a whole field of affordances. As shown in Figure 1, using a three-dimensional model, they assess the “width” which depict the breadth of the scope of affordances perceived, the “depth” which refers to the temporal aspect, and the “height” which signifies the importance of each affordance. By sketching the fields of relevant affordances, they noticed that psychologically healthy patients perceive a broad, deep and varied field of affordances, depressed patients perceive a homogenized field, while people suffering from Obsessive–Compulsive Disorder (OCD) perceive one very broad, deep, and high affordance, limiting the attention directed toward others in the field.

Assessing athletes’ perceived field of affordances could be a solution worth investigating to facilitate the person-environment fit. By questioning athletes on the possibilities for action they perceive are afforded in their sport environment, the degree of sustainability over time as well as the degree of significance of these possibilities in their overall life, it could be possible to track changes in their way of being in the (sport) world throughout their development. Building on the *enactive affordance-based model* (de Haan et al., 2013), athletes maintaining a broad, wide and varied field of affordances following specialization, for instance, could be a relevant indicator that they are interacting meaningfully with their environment (Figure 1A), suggesting that the person-environment fit is suitable to fulfill their holistic development. On the other hand, athletes presenting an unattractive (Figure 1B – nothing is perceived as significant) or unbalanced (Figure 1C – sport is hiding everything) field of affordances could indicate the need to further investigate how the athlete is experiencing the world in order to re-adjust various environmental constraints before it leads to aforementioned consequences such as drop in motivation, burnout, or dropout. In short, instead of tracking retrospectively number of training hours, effort invested, as well as relevance and enjoyment of practice activities, here, we argue that supporting and tracking ongoing interactions between athletes and their environments could be a promising way to support elite athlete holistic development. In this vein, Hauw (2018) suggest an *enactive approach* in sport psychology to help athletes balance their activities in and outside of sport to facilitate coping with sport perturbations (e.g., injuries, selections, and transitions). This approach is highly relevant to what we are suggesting, which deserves further investigation.

### Perspectives

To be acted on meaningfully, affordances must be perceived by an individual embedded within a form of life. If fact, if affordances are opportunities for actions, perspectives “may be understood broadly as perceptual and conceptual orientations to a situation with a view of acting within that situation” (Martin, 2005, p. 231). Going back to the evolution of practice design in sport, perception-action coupling is central to the emergence of versatile and adaptive movement (Araújo et al., 2019). One of the goals of the ecological dynamics approach is to guide an athletes’ attention toward a broad variety of opportunities for action (Renshaw et al., 2010; Araújo et al., 2019). Hence, the engagement in other sport activities to enrich athletes’ skill repertoire is encouraged (Strafford et al., 2018). For instance, “donor sports”, which are sport activities different from the targeted sport, are believed to “donate” certain athletic skills through various interactions with an affordance landscape that shares similarities with those experienced within the targeted sport (Rudd et al., 2020). To test this assumption, Parkour – initially develop as a “free running activity” requiring individuals to immerse themselves in “one’s immediate physical/natural environment to gain a deep phenomenological awareness of it” (Atkinson, 2009, p. 172) – is considered an excellent “donor sport” candidate (Strafford et al., 2018). It was shown that experienced Parkour Tracers develop skills such as multi-limb coordination, control precision, rate control, and response orientation through the variety of interactions sustained during practice (Strafford et al., 2020). Hence, when athletes from other sports engage in Parkour-style training, they are challenged to adaptively negotiate all sorts of obstacles that could be transferable to sport-specific contexts (see Strafford et al., 2018, for multiple examples).

Building on the donor sport idea, we argue that “donor activities”, defined here as activities outside the domain of sport, could help broaden an athletes’ perspective of the affordance landscape. After all, Glăveanu (2021) suggests that *difference* is what enables and cultivates perspective, and such difference could be achieved by exposing athletes to a variety of donor activities. Moreover, it is our contention that to evolve and support athletes’ holistic growth, the sport system must accept, embrace, and even encourage differences in athletes’ approaches toward excellence. For instance, rather than conceptualizing activities outside of sport as a distraction, or a barrier to the achievement of “optimal” training time, sport leaders may benefit from observing carefully what it *donates* to the athlete. How do athletes’ perspectives evolve when they go to school, engage in an internship or gain professional experiences? How does an athletes’ motivation or intention change when they are encouraged to pursue other interests?

In the scientific domain, the serious engagement in “donor activities”, or what is also called “integrated network of enterprise”, was found to be associated with scientific success as defined by Nobel prize awards (Root-Bernstein et al., 2008). Specifically, biographies of Nobel laureates demonstrate that they were more likely to be seriously engaged in various forms of arts and crafts than any other groups of scientists, challenging the conception that “the degree of energy and persistence required to gain ever-higher levels of success in science [is] inversely proportional to the amount of energy left over to explore avocational talents in arts and crafts” (Root-Bernstein et al., 2008, p. 56). Moreover, they showed that engagement in activity beyond those of the “disciplinary specialized” help broaden scientists’ perception of the world, which could benefit their scientific discovery: a finding supported by none other than Albert Einstein:

“The theory of relativity occurred to me by intuition, and music is the driving force behind this intuition […] My new discovery is the result of musical perception” (Quoted in Curtin, 1982, p. 84).

Some may argue that sport does not require the same level of creativity as does scientific discovery, limiting the application of such results to the athletic domains. Yet, if we move away from the conception of creativity as a product, a personality trait or a thought preceding action, and define it as a process that “both explores and expands the area of possibility for individuals, groups, and society” (Glăveanu, 2021, p. 17), engaging with all sorts of activities in and beyond sport may be beneficial (Richard et al., 2021). To some extent, we may even want to question whether restricting athletes possibilities to engage with their environment creatively for the benefit of mechanical and repeatable states of being in the world is ethical (Rasmussen et al., 2022). By remaining open and responsive to what other activities can “donate”, sport could become much more inclusive of differences. The power of perspectives derives “from their capacity to both guide individual actions and make joint action possible, while at the same time, opening us to the possible by endowing our actions with spontaneity, flexibility, and openness toward others and the future” (Glăveanu, 2021, p. 54).

### Meaningful actions and possibilities

What happens, then, when flexible perspectives meet a rich and significant field of affordances? According to Glăveanu’s (2021) socio-cultural theory, at the interaction between perspective and affordances lies meaningful actions. Actions can be considered both normative due to cultural influences, but also agentic when one embraces the freedom to act differently. In other words, while conventional affordances and hegemonic perspectives may limit actions and reinforce sameness, welcoming difference in perspectives in an environment affording varied opportunities encourage unique actions creating a space to explore *the possible*. The notion of “cultivating the possible” was central to the cognitive revolution led by Jerome Bruner initiating, in the 1960s, an education reform in the United States (Bruner, 1960). According to him, “the object of learning was to open up realms of possibility beyond just the sheer business of what you learn” (Bruner, 2012, p. 28). Inspired by Bruner’s theoretical perspectives, Glăveanu (2021) defined the possible as “both the awareness and exploration of the space created by developing multiple instead of singular relation with the world” (p. 3). Perhaps, then, the notion of *the possible* could also inspire a reform in sport?

**Figure 2:**
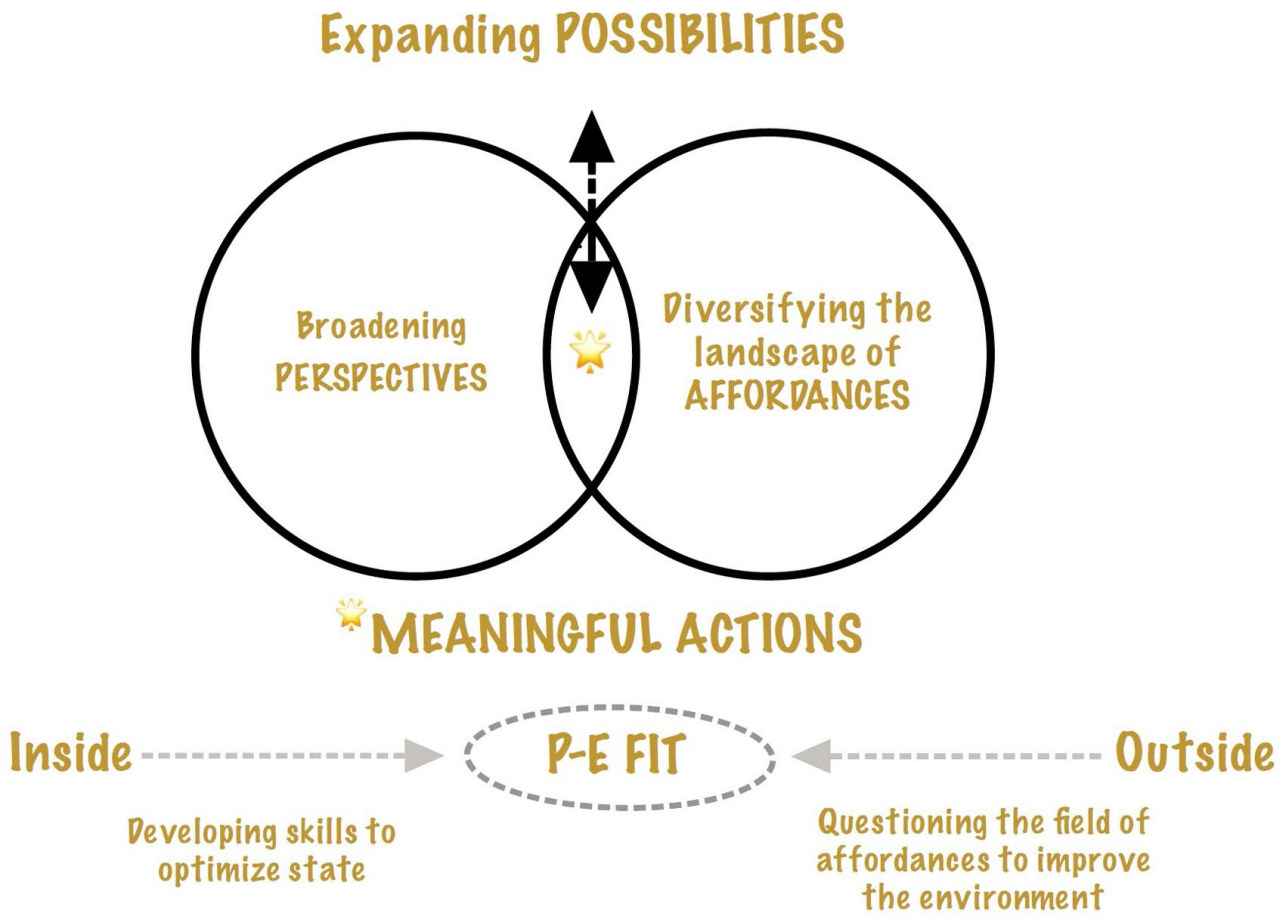
A Person–Environment (P–E) fit model of high-performance sport.

Figure 2 shows that instead of forcing rigid specialization, the sport system could encourage the exploration of a broad range of developmental possibilities, even at the highest level. To conceptualize specialization by building on the person-environment fit, athletes’ skill development (i.e., inside-out strategies) must be accompanied by outside-in strategies. That is, questioning which opportunities afforded by the sports form of life are significant for the athlete. Namely, understanding which activities, projects, and responsibilities attract them could help design a rich and diversified landscape of affordances where the athlete can explore, discover, and interact with their environments. To ensure they remain open and responsive to the richness of this landscape, accepting, integrating, and even stimulating differences may be crucial. Being surrounded by differences can broadened athletes’ perspectives by challenging psychological flexibility, curiosity and interpersonal skills (Glăveanu, 2021; Richard et al., 2021). As illustrated by the vein diagram in Figure 2, by diversifying the field of affordances and broadening perspectives, athletes can act meaningfully both in and outside of sport enhancing their sense of possibility. Exploring the possible is associated with creativity, psychological health, respect for otherness, as well as personal and societal growth (Glăveanu, 2021). Consequently, conceptualizing the outcome of specialization on the exploration and achievement of the possible rather than on the accumulation of medals may be a way to reconnect with the roots of Olympism; “improving and striving for perfection on a daily basis, both in the stadium *and in life*.”

### Changing the outcome

To transition from a restrictive specialization philosophy, to one that is open to possibilities, we must change the outcome measure. Currently, the great majority of studies examining environmental conditions at the highest-level define sport success in terms of medals and/or achievement. For example, they compare medalists with non-medalists (Güllich, 2017; Güllich et al., 2019) or Olympians versus non-Olympians (Law et al., 2007). While this could be considered an objective criterion to differentiate between successful and less-successful athletes, it could also be considered heavily biased by westernized socio-cultural norms and expectations rooted in materialism.

Baker et al. (2022) used contemporary evolutionary theory to challenge the way athletes’ long-term potential for success is currently evaluated. To reach the highest level in sport, athletes must go through selection processes which determines “who remains and who is removed from a sample of potential athletes” (p. 1). Usually, this process involves a series of “performance” tests most likely assessing physical and psychological attributes combined with coach or scout observations (Johnston and Baker, 2019). Because the sport system is complex, selection processes are influenced by “selection pressure”. That is, athletes must present either “survival advantages” or “attractive advantages” that suits the specific environments to be selected. While “survival advantages” refer to superior performance level at a certain point in time in a specific context (which could be biased by a myriad of factors like relative age effect), the “attractive advantages” refer to characteristics that selectors (e.g., coaches or scouts) appreciate and value. For instance, if work ethics, confidence, focus, and “coachability” is highly valued, while originality, risk taking, exuberance is dampened, it may lead to an over representation of the former characteristics in high-level athletes.

This holds true for the impact of specialization. If committing to high quantity of specialized training is what is valued by a sport system, it increases the likelihood that athletes who can comply and tolerate such a regime will make it to the top. Athletes that “fit” the system’s norms and expectations are selected, thereafter benefiting from superior resources, increasing their chances to be “successful”. In other words, the current measure of success (i.e., medals) may be a reflection of what is promoted (i.e., excessive and restrictive specialization). Meaning, specialization is not what led to success *per se*, but what is culturally accepted and rewarded.

By fostering an environment in which athletes can explore diverse affordances, late specialization may end up being just one (of many) pathway(s). The proposed shift toward possibilities may allow us to better capture the complexity of athletes’ development. By diversifying the “offer”, sport could attract and retain a broader variety of athletic potential. To achieve this, though, potential realization cannot only equate winning an Olympic medal. A study examining the success narratives of Olympic and elite athletes provides a relevant framework to initiate the broadening of the definition of success in sport (Carless and Douglas, 2012). Specifically, findings revealed that, beyond performance narratives, elite athletes were defining success in terms of embodiment and discovery (e.g., the simple pleasure of performing specific sport movements), effort and application (e.g., recognizing that they controlled what they could) and relationships (e.g., the people they encounter through their journey). These narratives helped those athletes resist the dominance of the performance narrative culture allowing them to remain healthy, sustain involvement, and maintain performance. By integrating health, wellbeing, and fulfillment in addition to performance as indicators of success it may completely change the landscape of who is considered “successful”.

### Theory into practice

To illustrate how this conceptualization of specialization can be articulated in high performance sport beyond our theorizing, we will now integrate an example of how a “highly talented” athlete, whose motivation dropped importantly after a few years of specialization in an individual sport, re-connected with their sport through exposure to donor activities. This example is rooted in the first authors experience working in high-performance sport. Following a few unsuccessful attempts to “fix” the athlete’s motivation through “inside out” strategies, such as goal setting and emotional regulation, a near qualification miss for a spot to compete at the Olympics pushed the sport organization to reconsider their talent development approach. Through conversations with the athlete, it was identified that the rigid requirement of specialization was preventing them to invest time in the pursuit of a significant artistic career goal, which made the athlete question their commitment to sport. In other words, the athlete’s perceived field of affordance was assessed as unbalanced, with their artistic interest being hidden by a single broad, deep and high sport affordance. To re-balance the environment, the sport organization first accepted the athlete’s request to take an extensive break from sport (without losing funding) to explore their artistic interest. This exploration allowed the athlete to act meaningfully on artistic affordances outside of the sport form of life, leading to the realization that, with a few adjustments, it could be possible to pursue *both* careers. Acting on different affordances also made them gain perspective over their sport engagement. For the first time, they realized that the physical and psychological challenges afforded by their sport were significant for them. Following the break, the training requirements were flexibly co-designed so the athlete could actively participate in artistic “gigs” when the opportunities presented. The freedom to act on artistic affordances, while pursuing high-level training in sport, resulted in an expanded field of possibilities. Sport, in other words, was no longer perceived as a barrier to their artistic pursuit, but as a *complementary possibility to grow*. Opening spaces for this athlete to act on multiple affordances resulted in enhanced desire to train, compete, and excel in and outside of sport.

Given prescriptively rigid systems are oft-culturally well anchored in sport, a shift toward expanding possibilities for athletes at the highest level may appear impossible if they want to put all chances on their side to succeed. Yet, it is hard to claim with certainty that the attraction and pursuit of multiple interests harms performance at the highest level as this is rarely permitted by the system and not frequently researched. For instance, to “chase other dreams”,^1^ the world No.1 tennis player and three-time Grand Slam winner Ashleigh Barty, decided to step away from the physical and psychological demands of the sport environment by retiring. While there is much to applaud in the decision to retire while still at the “top”, the question of whether such decision would be different in a more flexible and permissive system is intriguing. Answering this question through the theorizing presented in this paper is in itself a promising affordance that in its realization could create sustainable changes for athletes in high-performance sport.

## Conclusion

Olympism has become a multi-billion-dollar entertainment industry (Rees et al., 2016), and each medal has its price tag. Perhaps this explains, in-part, why many have lost sight of the holistic developmental goals? Is Olympism, in other words, only about lucrative entertainment, or could it also be more than that? By conceptually shifting the notion of specialization from one of quantity to one of possibility, we argued that high-performance sport can lead to holistic development. Instead of pre-establishing rigid training requirements that everyone must adhere to, we suggested that elite athletes could be provided with opportunities to explore what is meaningful for them to thrive beyond their sport. Indeed, while some may end up specializing in sport, some may choose to diversify their activities. A person-environment fit, thus, welcomes all approaches, so long as it sustains the person’s health, wellbeing, and performance.

Because no gold medal justifies poor health and wellbeing, it is time for elite sport to rethink their success criteria. As brilliantly stated by Whitehead and Senecal (2020): “[i]t is assumed that winning feels good, but how *does* it feel at the end of the day? The Olympic gold medalist does not somehow win at development. A chariot does not descend from Mount Olympus to scoop them up. The athlete must still navigate difficult issues of well-being, purpose, and meaning in occupation and family as they emerge throughout the rest of their lives” (p. 157). By expanding rather than reducing developmental possibilities, sport may hold open spaces and provide a sustainable environment in which athletes can continue to find ways of *carrying on*.

## Author contributions

VR, JC, and CW contributed to the conception and design of the manuscript and models. VR conducted the literature review and led the drafting of the manuscript. CW provided theoretical critique and general direction. JC provided high-level commentary. Each version of the manuscript was revised by the group adding significant contributions to the content of specific sections. All authors approved the submitted version.

## Conflict of interest

The authors declare that the research was conducted in the absence of any commercial or financial relationships that could be construed as a potential conflict of interest.

## Publisher’s note

All claims expressed in this article are solely those of the authors and do not necessarily represent those of their affiliated organizations, or those of the publisher, the editors and the reviewers. Any product that may be evaluated in this article, or claim that may be made by its manufacturer, is not guaranteed or endorsed by the publisher.

## Footnotes

^1^For the full interview "World No 1 Ash Barty announces shock retirement from tennis in Instagram post" visit the Guardian Sport YouTube channel at: https://www.youtube.com/watch?v=CgpwtlUHBRI&ab_channel=GuardianSport
